# Three cases of histologically proven hepatic epithelioid hemangioendothelioma evaluated using a second-generation microbubble contrast medium in ultrasonography: case reports

**DOI:** 10.1186/s12876-019-1113-y

**Published:** 2019-11-14

**Authors:** Jun Arai, Yuu Shimozuma, Yumi Otoyama, Ikuya Sugiura, Yoko Nakajima, Eiichi Hayashi, Atsushi Kajiwara, Risa Omori, Shojiro Uozumi, Miyuki Miyashita, Manabu Uchikoshi, Hiroyoshi Doi, Masashi Sakaki, Tianpeng Wang, Junichi Eguchi, Takayoshi Ito, Toshikazu Kurihara, Jiro Munechika, Takehiko Gokan, Koji Saito, Sakiko Miura, Genshu Tate, Masafumi Takimoto, Hitoshi Yoshida

**Affiliations:** 1 Department of Medicine, Division of Gastroenterology, Showa University School of Medicine, Tokyo, Japan; 2 Digestive Disease Center, Showa University Koto Toyosu Hospital, Tokyo, Japan; 3 Internal Medicine, Showa University Karasuyama Hospital, Tokyo, Japan; 4 Division of Radiology, Department of Medicine, Showa University School of Medicine, Tokyo, Japan; 5 Division of Pathology, Department of Medicine, Teikyo University Hospital, Tokyo, Japan; 6 Division of Pathology, Department of Medicine, Showa University School of Medicine, Tokyo, Japan

**Keywords:** Hepatocellular carcinoma, Epithelioid Hemangioendothelioma, Perfusion imaging, Sonazoid®

## Abstract

**Background:**

Hepatic epithelioid hemangioendothelioma (HEH) is rare; it is reported in < 1 person in 1,000,000 individuals. For accurate diagnosis, information regarding multiple graphic modalities in HEH is required. However, there is very little information concerning Sonazoid® contrast enhanced ultrasonography (CEUS) in HEH.

**Case presentation:**

The present report describes the histologically proven three HEH cases evaluated using Sonazoid® CEUS. Case 1 was a 33-year-old female patient with no relevant past medical history, who experienced right upper quadrant pain. Conventional abdominal US revealed multiple low echoic liver nodules with vague borderlines. In CEUS, the vascularity of the nodules was similar to that seen in the neighboring normal liver. Later in the portal venous and late phases (PVLP) and post vascular phase, washout of Sonazoid® was detected in the nodules. Case 2 was a 93-year-old female patient with a previous medical history including operations for breast cancer and ovary cancer in her 50’s. Conventional abdominal US revealed multiple low echoic nodules, some of which contained cystic lesions. In the early vascular phase of CEUS, nodules excluding the central anechoic regions were enhanced from peripheral sites. Although the enhancement inside the nodules persisted in both the PVLP and post vascular phase, anechoic areas in the center of some nodules were not enhanced at all. Case 3 was a 39-year-old male patient presented with right upper-quadrant pain, without any relevant past medical history. Conventional abdominal US revealed multiple low echoic liver nodules. In the early vascular phase of CEUS, nodules were gradually enhanced from the peripheral sites as ringed enhancement. Sonazoid®was washed out from the nodules in the PVLP and post vascular phase.

**Conclusions:**

The most important feature was peripheral enhancement in the early vascular phase. In case 2, the enhancement of the parenchyma of liver nodules persisted even in the PVLP; indicating the lower degree of malignant potential than others. Actually, the tumors did not extend without any treatment in case 2. Since case 2 is the first case report of HEH with cystic lesions, in patients with liver nodules including cystic lesions, HEH is a potential diagnosis.

## Background

The prevalence of hepatic epithelioid hemangioendothelioma (HEH) is low; it is reported in < 1 person in 1,000,000 individuals [1]. In Japan, HEH was first reported in 1982 [2]. In a survey of 63 HEH cases in the Japanese population, the average patient age was 48 (range, 16–82) years [3]. Earnest et al. retrospectively investigated a cohort of 96 patients and reported that the prevalence of HEH peaked among patients aged 30–40 years [4]. We encountered three cases of HEH diagnosed from 2011 to 2015 at the Showa University Hospital. Graphical hallmarks of HEH in computed tomography (CT) or magnetic resonance imaging (MRI) have been reported [5]. However, there is very little information concerning Sonazoid® contrast enhanced ultrasonography (CEUS) in HEH. Sonazoid® is a second-generation microbubble contrast medium used in CEUS for visualizing the vascular pattern inside liver nodules [6–8].

The advantageous features of Sonazoid® CEUS include the correct diagnosis of not only hypervascular liver nodules such as hepatocellular carcinoma (HCC) [6, 9], but also hypovascular liver nodules [10]. In this study, we introduce the hallmark features of Sonazoid® CEUS in HEH, using the Toshiba US system.

A bolus of Sonazoid® suspension was intravenously injected, and in 15–30 s, the early vascular phase of the hepatic artery was visualized. Thereafter, from 30 s to 2 min after injecting, the information regarding hepatic tissue perfusion, namely the portal venous and late phases (PVLP), was recorded. Lastly in 10 min after injection, the post vascular phase was defined as the parenchymal finding.

The pathologic diagnosis involving hematoxylin and eosin-stained tumor sections and immunohistochemical staining was performed by experienced pathologists. This study was approved by a suitably constituted Ethics Committee of our institution (approval number is 1551128) and it complied with the provisions of the Declaration of Helsinki. The patients’ written informed consent was obtained for the publication.

## Case presentation

**Fig. 1:**
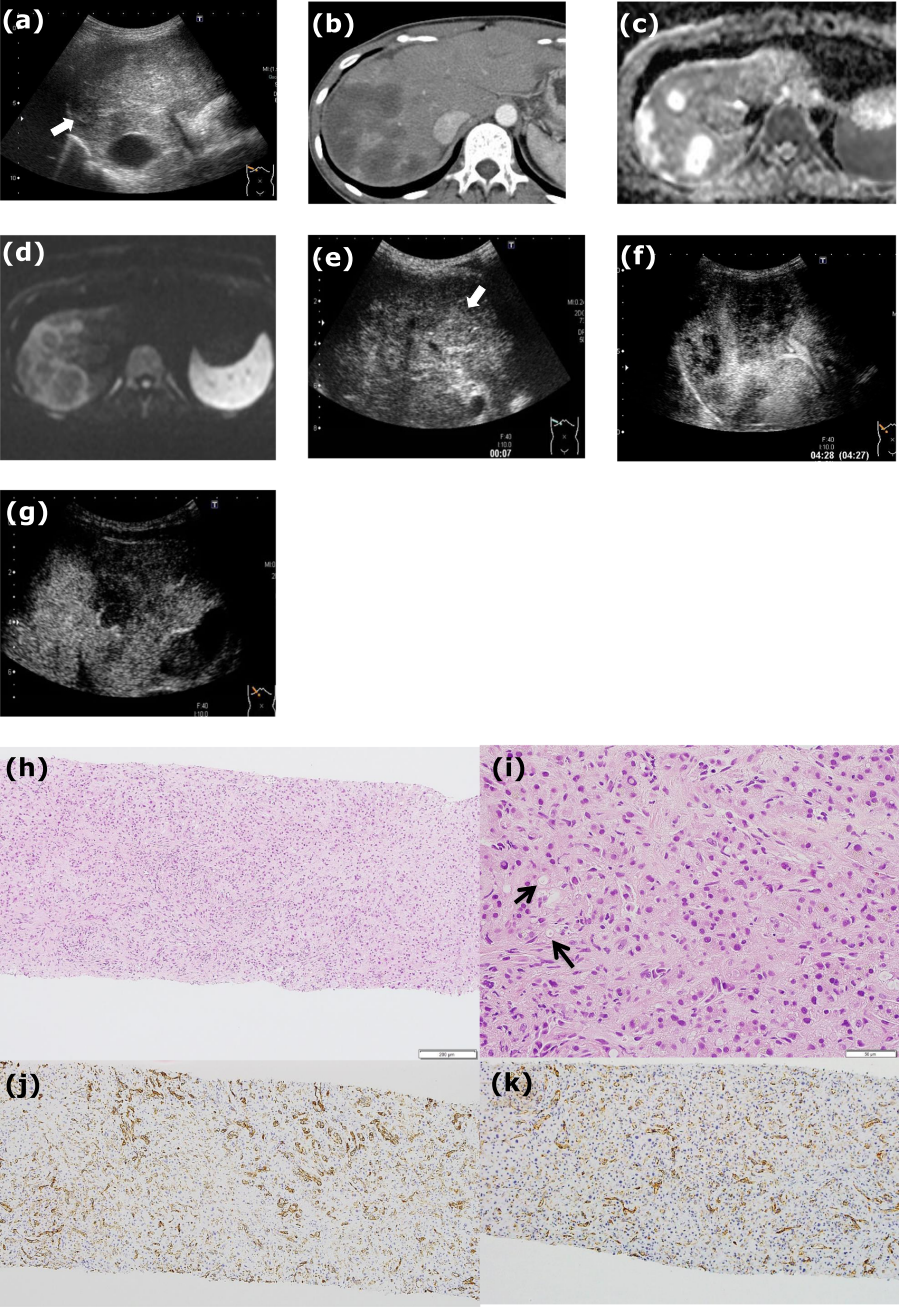
**a** Conventional abdominal ultrasonography (US) in case 1 showed multiple low echoic liver nodules (*arrow*) with a vague margin, and nodules located in the peripheral sites tended to coalesce with each other. The vascular pattern inside the nodules was not precisely visible in the arterial dominant phase of contrast-enhanced computed tomography (CECT) (**b**). **c** The apparent diffusion coefficient (ADC) mapping in case1 is shown. **d** In the diffusion-weighted imaging (DWI) at a b score of 1000 (sec/mm^2^), the peripheral area of the nodules in case1 showed a much higher intensity than its central lesion. **e** Sonazoid^**Ⓡ**^ contrast enhanced US (CEUS) in case1 showed the vascularity (*arrow*) in the early vascular phase, but the vascular pattern is not specified. **f** Later in the portal venous and late phases (PVLP) and post vascular phase, they were defective. **g** Defect re-perfusion imaging clearly showed that the nodules were gradually enhanced from the peripheral sites as ringed enhancement. **h** Photomicrograph of a histological section of a hepatic specimen obtained via percutaneous liver needle biopsy in case 1 (200× with hematoxylin and eosin stain) is shown. **i** A high number of epithelioid tumor cells with spindle-shaped nuclei, form intracellular vascular lumina (*arrow*) (800× with hematoxylin and eosin stain). In immunostaining (200×), the sample was positive for cluster of differentiation (CD) 31 (**j**) and CD34 (**k**)

Case 1 was a 33-year-old Japanese female patient with no relevant past medical history, who experienced right upper quadrant pain in 2011. Blood examination revealed slightly elevated gamma-glutamyltranspeptidase (γ-GTP) and C reactive protein (CRP) levels; carcinogen embryonic antigen (CEA), carbohydrate antigen 19–9 (CA19–9), *α*-fetoprotein (AFP), protein induced by vitamin K deficiency or antagonists-II (PIVKA-II), and HbA1c (National Glycohemoglobin Standardization Program; NGSP) levels were all normal. Hepatitis B virus surface (HBs) antigen and anti-hepatitis C virus (HCV) antibody were negative. Conventional abdominal US revealed multiple low echoic liver nodules with vague borderlines, and nodules located in the peripheral sites were attached adjacent to each other (Fig. 1a). CECT (contrast enhanced CT) revealed multiple low-density liver nodules in the arterial dominant phase, suggesting hypovascular tumors (Fig. 1b). The graphics in abdominal plain CT (Additional file 1: Figure S1a) and the equilibrium phase of CECT (Additional file 1: Figure S1b) are also shown. The chest CT (Additional file 1: Figure S1c) revealed multiple lung nodules. The lesions in the liver exhibited high signal intensity on axial T2-weighted imaging (T2WI); the central areas of these lesions had a much higher intensity (Additional file 1: Figure S1d), which was also seen using the apparent diffusion coefficient (ADC) map. Hyperintense signals were found in the peripheral lesions of nodules, whereas central lesions had hypointense signals in diffusion-weighted imaging (DWI) with b-factor of 1000 (sec/mm^2^) (Fig. 1d). The findings of the gadolinium ethoxybenzyl diethylenetriaminepentaacetic acid (Gd-EOB-DTPA) in the arterial dominant phase and the hepatocellular phase are shown in Additional file 1: Figure S1e and f, respectively. Gastrointestinal endoscopy and colonoscopy showed no evidence of advanced malignant tumors causing metastatic liver tumors.

**Fig. 2:**
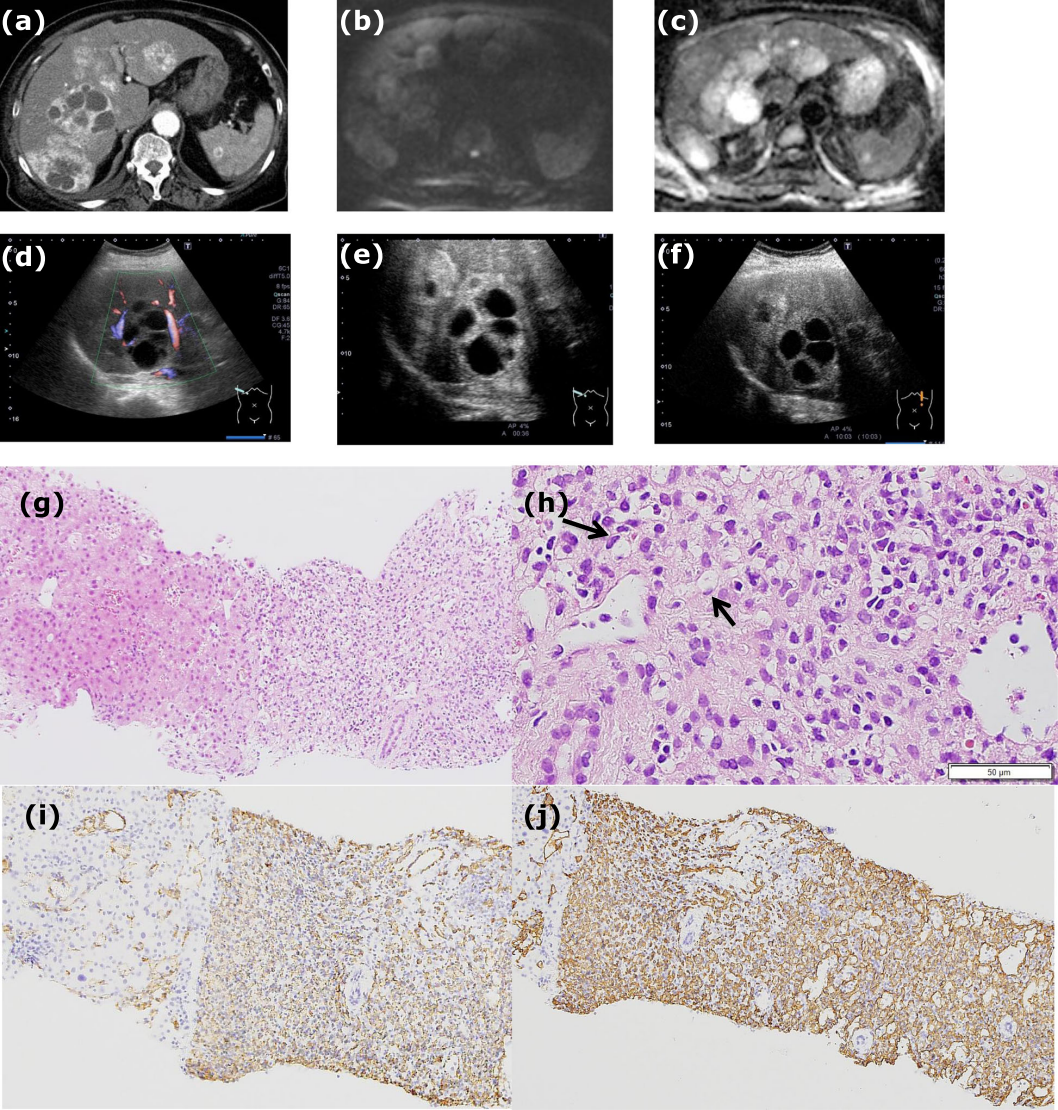
**a** The CT scan in case 2 showed multiple hypodense liver nodules, including cystic lesions, and enhancement inside the tumors started in peripheral lesions in the arterial dominant phase. **b** In the DWI, at a b score of 1000 (sec/mm^2^) the peripheral area of the nodules showed a much higher intensity than its central lesion. **c** ADC mapping is shown. **d** In the conventional abdominal US of case2, there are multiple low echoic nodules, some of which contained cystic lesions. Color flow signals were found in color Doppler imaging. In CEUS, the enhancement inside the nodules was found in both (**e**) the PVLP and (**f**) post vascular phase, whereas anechoic areas in the inner portion were not enhanced. **g** Photomicrograph of a histological section of a hepatic specimen obtained via percutaneous liver needle biopsy in case 2 (200× with hematoxylin and eosin stain) is shown. **h** A high number of epithelioid tumor cells with spindle-shaped nuclei, form intracellular vascular lumina (*arrow*) (800× with hematoxylin and eosin stain). In immunostaining (200×), the sample was positive for cluster of differentiation (CD) 31 (**i**) and CD34 (**j**)

Case 2 was a 93-year-old Japanese female patient with a previous medical history including operations for breast cancer and ovary cancer in her 50’s; she had epigastric tenderness, high fever, and jaundice. The patient presented at a community hospital and was referred to our hospital for further examination in 2013. On admission, the patient’s body temperature was 37.8 °C, with slightly elevated transaminases, γ-GTP, CRP, and CA19–9 (91.2 U/mL) levels, while levels of CEA, AFP, PIVKA-II, and HbA1c (NGSP) were normal. HBs antigen and anti-HCV antibody were negative. The noncontrast CT revealed multiple hypodense liver nodules, with cystic lesions inside them (Additional file 1: Figure S2a). In the arterial dominant phase, enhancement seemed to originate from the peripheral portions (Fig. 2a), and persisted in the equilibrium phase (Additional file 1: Figure S2b). The multiple nodules in the spleen were also detected (Additional file 1: Figure S2c, d, and e). T2WI showed multiple hyperintense liver nodules (Additional file 1: Figure S2c). In the DWI scans, the peripheral portions had a much higher intensity at 1000 (sec/mm^2^) b-factor (Fig. 2b). On the ADC map, hypointense signals were found in the peripheral lesions of the nodules, whereas central lesions had hyperintense signals (Fig. 2c). After the patient recovered from cholangitis, the CA19–9 level was normalized at 6.4 U/mL. Conventional abdominal US revealed multiple low echoic nodules, some of which contained cystic lesions (Fig. 2d). Gastrointestinal endoscopy revealed no evidence of advanced malignant tumors.

**Fig. 3:**
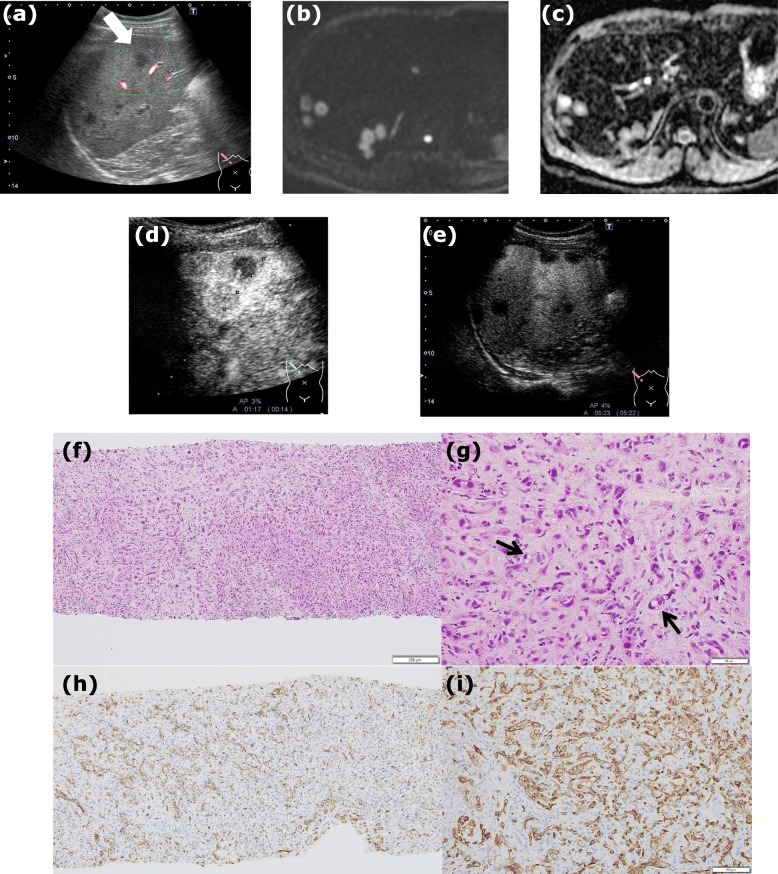
**a** In the conventional abdominal US in case 3, there were multiple low echoic liver nodules, predominantly seen in the right lobe (*arrow*). **b** In the DWI at a b score of 1000 (sec/mm^2^), the peripheral site of the nodules showed a much higher intensity than its central lesion. **c** ADC mapping is shown. **d** The early vascular phase of CEUS in case3 showed that the tumors were gradually enhanced from the peripheral sites. **e** In the PVLP and post vascular phase, they were defective. **f** Photomicrograph of a histological section of a hepatic specimen obtained via percutaneous liver needle biopsy in case 3 (200× with hematoxylin and eosin stain) is shown. **g** A high number of epithelioid tumor cells with spindle-shaped nuclei, form intracellular vascular lumina (*arrow*) (800× with hematoxylin and eosin stain). In immunostaining (200×), the sample was positive for cluster of differentiation (CD) 31 (**h**) and CD34 (**i**)

Case 3 was a 39-year-old Japanese male patient, without any relevant past medical history. He started to feel right upper-quadrant pain in 2015. CEA, CA19–9, AFP, and PIVKA-II tumor markers were normal, and HBs antigen and anti-HCV antibody were negative. Conventional abdominal US revealed multiple low echoic liver nodules; they were predominantly seen in the right lobe (Fig. 3a). The findings of the plain CT, CECT, and abdominal MRI (Additional file 1: Figure S3a, b, c, d) in case 3 were similar to those of case 1. On DWI, the peripheral lesions showed a much higher intensity at 1000 (sec/mm^2^) b-factor (Fig. 3b), while central portions had higher signal intensity on the ADC map (Fig. 3c). To rule out metastatic liver tumors, gastrointestinal endoscopy and colonoscopy were performed; they showed no evidence of advanced malignant tumors.

Finally, percutaneous liver needle biopsy was performed in all of these three cases; the tissues were composed of a high number of dense epithelioid-like tumor cells with spindle-shaped nuclei, forming intracellular vascular lumina (Figs. 1h, i, 2g, h, 3f and g). Since the tumor cells were positive for factor VIII (Additional file 1: Figure S1g, Figure S2f, and Figure S3f), cluster of differentiation (CD) 31 (Figs. 1j, 2i, and 3h), and CD34 (Figs. 1k, 2j, and 3i), it was thought that they originated from endothelial cells. There were no high-grade cell atypia, enlarged vessels, or peliosis-like lesions, which supported a diagnosis of hemangiosarcoma; therefore, the tumor was finally diagnosed as HEH. The clinical course of each case is shown in Table 1. Briefly, in case1, the patient chose to undergo liver transplantation and introduced to other university hospital. No further information of the patient was obtained after that. In case 2, the patient did not decide to take any treatment and carefully has been followed up at our outpatient clinic. Fortunately, the tumors have not enlarged at all with CECT (Additional file 1: Figure S2d and S2e) when approximately 70 months has passes since the diagnosis was made. In case 3, the patient was treated with Adriamycin and Ifomide for four months and has been regularly followed up at our outpatient clinic without any further treatment. The tumors have not enlarged in the evaluation with CECT when approximately 49 months has passes since the diagnosis was made (Additional file 1: Figure S3e).

**Table 1:**
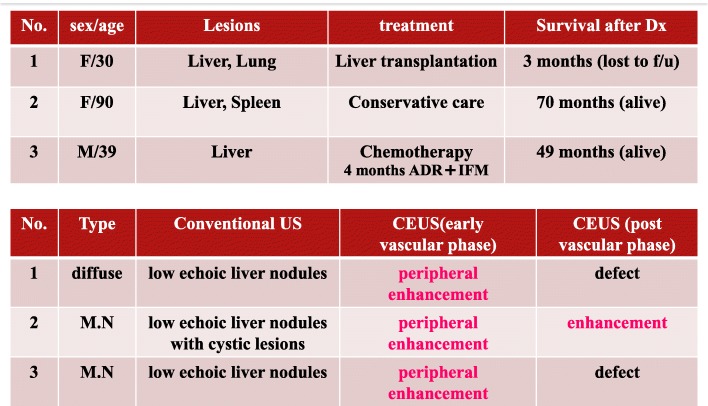
The clinical course of each case is shown. Abbreviations are written below

*Dx* diagnosis, *f/u* follow-up, *M.N* multifocal nodular type, *ADR* Adriamycin, *IFM* Ifomide

In all three cases, we performed Sonazoid® CEUS before percutaneous liver needle biopsy was performed. In the early vascular phase of case 1, the vascularity of the nodules was similar to that seen in the neighboring normal liver, but the vascular pattern was not specified (Fig. 1e). Later in the PVLP and post vascular phase, washout of Sonazoid® was detected in the nodules (Fig. 1f). Use of the defect re-perfusion imaging method involving repeat injection of Sonazoid® clearly showed that nodules were gradually enhanced from the peripheral sites as ringed enhancement (Fig. 1g). In the early vascular phase of case 2, nodules excluding the central anechoic regions were enhanced from peripheral sites. Although the enhancement inside the nodules persisted in both the PVLP (Fig. 2e) and post vascular phase (Fig. 2f), anechoic areas in the center of some nodules were not enhanced at all. In the early vascular phase of case 3, nodules were gradually enhanced from the peripheral sites as ringed enhancement (Fig. 3d). Sonazoid®was washed out from the nodules in the PVLP and post vascular phase (Fig. 3e).

## Discussion and conclusions

HEH has a moderate level of malignancy; therefore, it might result in a relatively poor prognosis if not correctly diagnosed at the right time [11]. However, the correct diagnosis of HEH generally takes from 3 months to 2 years after being symptomatic [12]. Therefore, the collection of useful information for HEH in various modalities is required. HEH is categorized as follows: 1) the single nodular type; 2) the multifocal nodular type; and 3) the diffuse type [13]. Cases 1 and 3 matched to the features of type 2). However, case 2, which contained cystic lesions that were anechoic (Fig. 2d), could not be categorized into any of the groups mentioned above. In previous studies, numerous HEH cases with moderately hypoechoic lesions in the center of nodules have been reported. They are generally thought to be necrosis or focal hemorrhages [14, 15], but are never anechoic. As a tumor progresses, it could possess necrotic tissues inside it; the borderline between the tumor cells and necrotic tissues is often vague. However, in case 2, the cystic lesion was clearly delineated from the tumor parenchyma using CEUS as well as using CECT (Fig. 2a, e). As far as we have investigated, there has been only one case report with multiple small liver cysts of fluid density in CT; however, other clinical or radiographic features related to that case were not introduced at all [12]. Therefore, case 2 is the first report of HEH including cystic lesions to fully introduce its clinical or radiographic findings. Cases similar to case 2 need to be reported in order to collect sufficient new information to categorize this type of HEH correctly. Recently, Dong et al. reported that the features of CEUS using SonoVue® enhancement in HEH involve a rim-like or heterogeneous hyperenhancement in the arterial phase and hypoenhancement in the PVLP [13].

Although there is very little information concerning Sonazoid® CEUS in HEH, the hallmark features of Sonazoid® CEUS concerning HEH could be the gradual enhancement from the peripheral sites in the vascular phase in these three cases. This pattern resembles that in hemangioma, which has already been reported as peripheral nodular enhancement with gradual centripetal filling [16]. However, there were no other cases in which liver needle biopsy was performed with a suspected diagnosis of HEH in my affiliation.

On an ADC map, hypointense signals in the peripheral lesions of nodules and hyperintense signals in the central lesions were found in all three cases. Other groups have previously reported that CEUS can differentiate between benign and malignant liver lesions by analyzing the portal venous phase [17, 18]. In Sonazoid® CEUS, although the feature of hypoenhancement in PVLP and the defect in the post vascular phase was detected in cases 1 and 3, in case 2 the enhancing effect persisted in the parenchyma even in the post vascular phase. This result is different from the previous reports concerning CEUS in HEH [13]. In our clinical experiences of three cases, hypoenhancement in PVLP and defects in the post vascular phase using CEUS, which could be clinically significant findings of malignancy, was seen in case 1 and case 3. Actually, the hepatic tumors have not enlarged at all in case 2 even if the patient did not take any medical treatment, which was concurrent with the lower malignant potential predicted using the findings from CEUS. However, the lost follow-up of the case1 is a limitation of this case report. In addition, the golden standard to investigate the contribution of some graphical findings to right diagnosis is describing a ROC curve. But it is impossible here because of its small number of case reports.

Although an accurate diagnosis of HEH takes considerable time, HEH is one of the important differential diagnoses for liver tumors, especially in patients without medical histories including chronic liver diseases or proceeding primary cancers.

In conclusion, to make an accurate diagnosis, the most important feature of these three cases could be peripheral enhancement using Sonazoid® CEUS in the early vascular phase; this highlights the need to suspect HEH, even when the liver nodules contain cystic anechoic lesions. The features of CEUS in PVLP might predict its malignant potential.

## Supplementary information

**Additional file 1: Figure S1.** (a) The graphics in abdominal plain CT and (b) the CECT equilibrium phase of case1 are shown. (c) The chest CT of case1 also revealed multiple lung nodules (*arrow*). (d) The lesions in the liver of case1 exhibited high signal intensity on axial T2WI. (e) The arterial dominant phase and (f) the hepatocellular phase in Gd-EOB-DTPA are shown. (g) In immunostaining (200×), a histological section of a hepatic specimen obtained via percutaneous liver needle biopsy in case 1 was positive for factor VIII. **Figure S2.** (a) The noncontrast CT of case2 revealed multiple hypodense liver nodules, with cystic lesions. (b) In the equilibrium phase of CECT, enhancement persisted inside the tumors. (c) T2WI showed multiple hyperintense liver nodules. (d) The size of liver tumors in the arterial dominant phase of CECT and (e) in the equilibrium phase have not increased when approximately 70 months has passes after the diagnosis was made. (f) In immunostaining (200×), a histological section of a hepatic specimen obtained via percutaneous liver needle biopsy in case 2 was positive for factor VIII. **Figure S3.** (a) The noncontrast CT of case3 revealed multiple hypodense liver nodules. (b) The graphics of abdominal CT in the arterial dominant phase and (c) in the equilibrium phase are shown. (d) The arterial dominant phase in Gd-EOB-DTPA is shown. (e) The size of liver tumors of case3 in the arterial dominant phase of CECT have not increased when approximately 49 months has passes after the diagnosis was made. (f) In immunostaining (200×), a histological section of a hepatic specimen obtained via percutaneous liver needle biopsy in case 3 was positive for factor VIII.

## Data Availability

Not applicable.

## Abbreviations

ADC: Apparent diffusion coefficient
AFP: *α*-fetoprotein
CA19–9: Carbohydrate antigen 19–9
CD: Cluster of differentiation
CEA: Carcinogen embryonic antigen
CEUS: Contrast enhanced ultrasonography
CRP: C reactive protein
CT: Computed tomography
DWI: Diffusion-weighted imaging
Gd-EOB-DTPA: gadolinium ethoxybenzyl diethylenetriaminepentaacetic acid
HBs: Hepatitis B virus surface
HCC: Hepatocellular carcinoma
HCV: Hepatitis C virus
HEH: Hepatic epithelioid hemangioendothelioma
MRI: Magnetic resonance imaging
NGSP: National Glycohemoglobin Standardization Program
PIVKA-II: Protein induced by vitamin K deficiency or antagonists-II
PVLP: Portal venous and late phases
T2WI: T2-weighted imaging
γ-GTP: gamma-glutamyltranspeptidase
