# Supplementary figures and images for: Three cases of histologically proven hepatic epithelioid hemangioendothelioma evaluated using a second-generation microbubble contrast medium in ultrasonography: case reports
Source: BMC Gastroenterol. 2019 Nov 14;19:187. doi: 10.1186/s12876-019-1113-y (PMC6857163; doi:10.1186/s12876-019-1113-y)

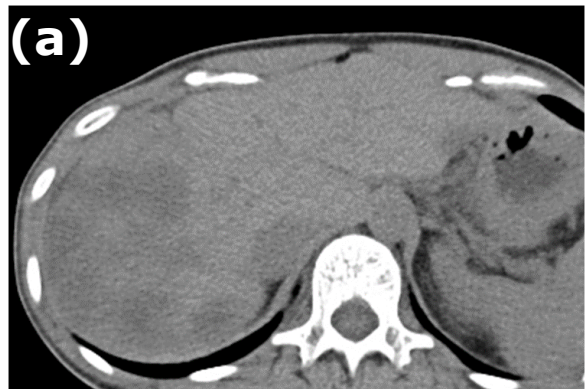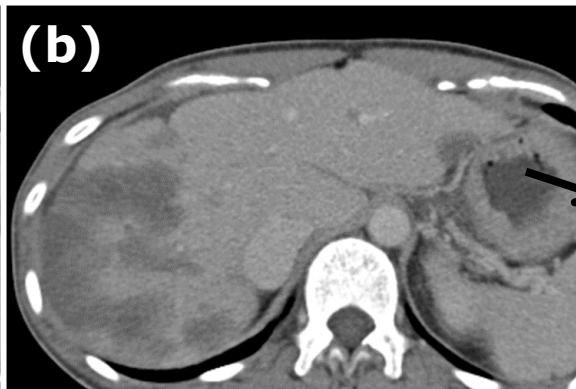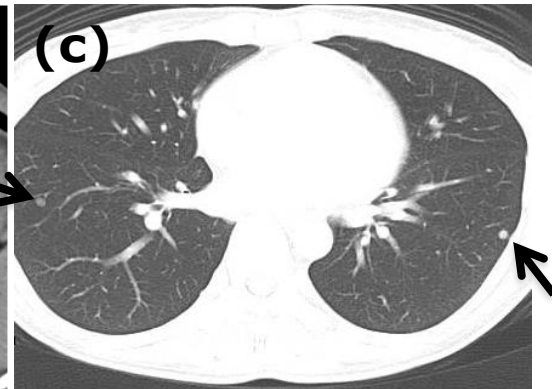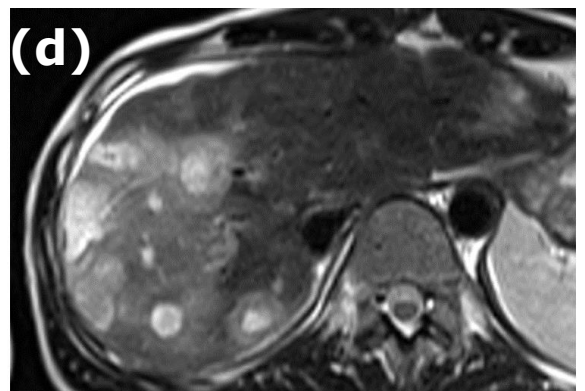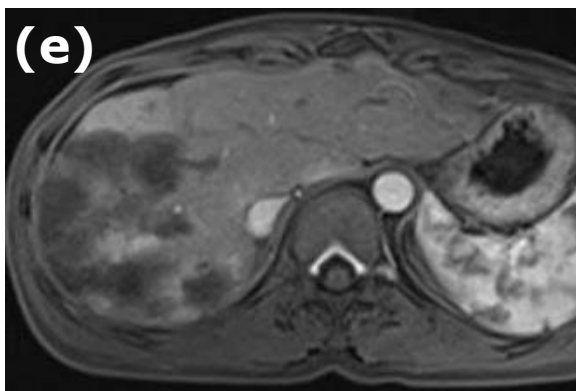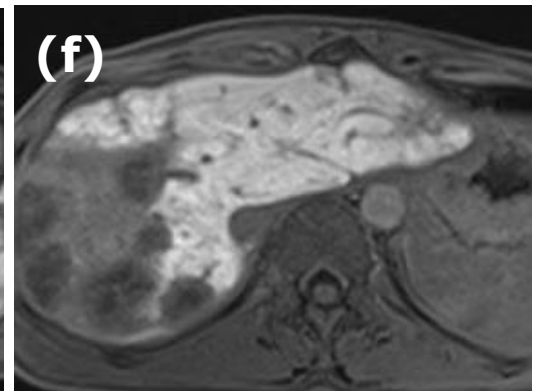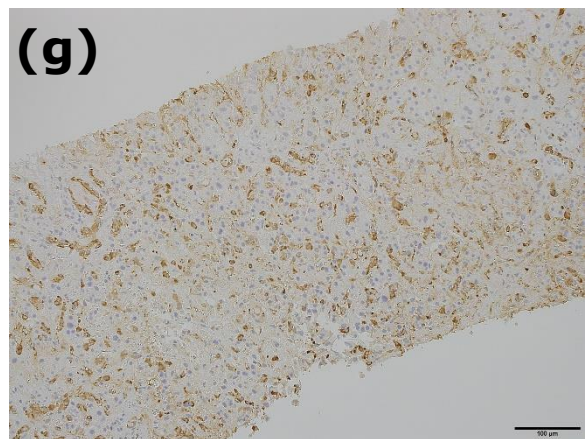

**Supple. Figure 1**

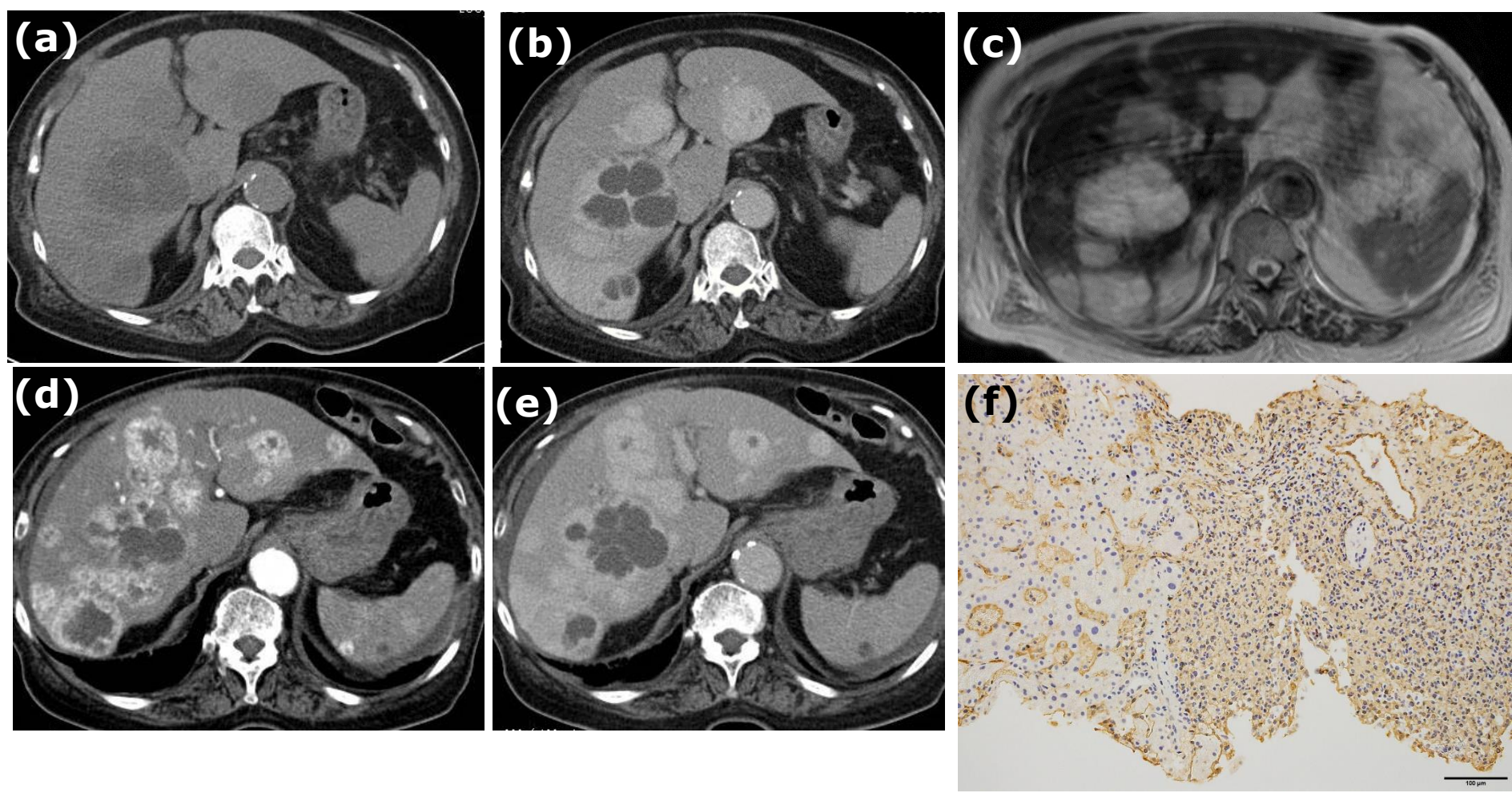

**Supple. Figure 2**

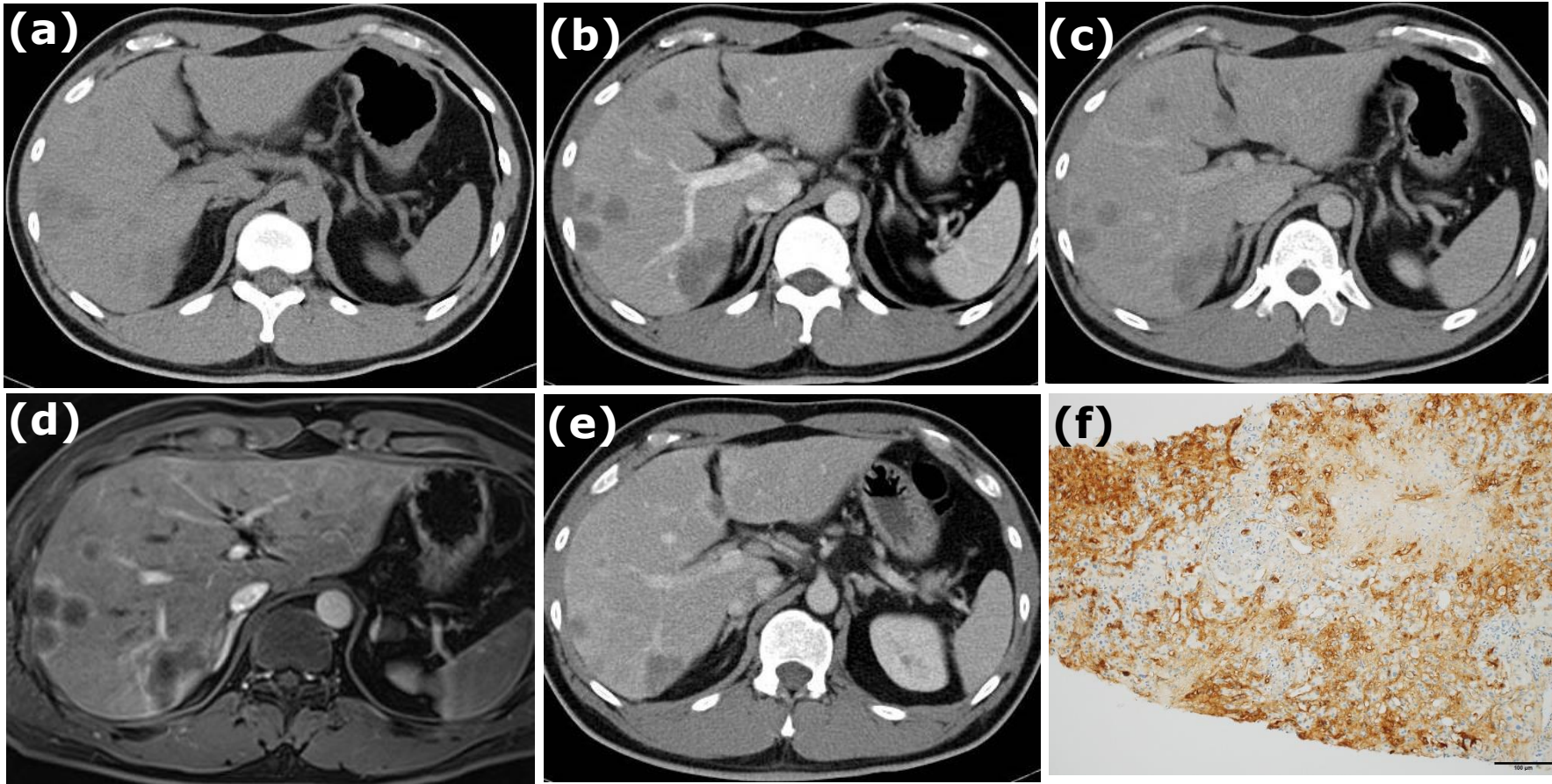

**Supple. Figure 3**

Supplement: Supplementary file 1 — Additional file 1: Figure S1. (a) The graphics in abdominal plain CT and (b) the CECT equilibrium phase of case1 are shown. (c) The chest CT of case1 also revealed multiple lung nodules (arrow). (d) The lesions in the liver of case1 exhibited high signal intensity on axial T2WI. (e) The arterial dominant phase and (f) the hepatocellular phase in Gd-EOB-DTPA are shown. (g) In immunostaining (200×), a histological section of a hepatic specimen obtained via percutaneous liver needle biopsy in case 1 was positive for factor VIII. Figure S2. (a) The noncontrast CT of case2 revealed multiple hypodense liver nodules, with cystic lesions. (b) In the equilibrium phase of CECT, enhancement persisted inside the tumors. (c) T2WI showed multiple hyperintense liver nodules. (d) The size of liver tumors in the arterial dominant phase of CECT and (e) in the equilibrium phase have not increased when approximately 70 months has passes after the diagnosis was made. (f) In immunostaining (200×), a histological section of a hepatic specimen obtained via percutaneous liver needle biopsy in case 2 was positive for factor VIII. Figure S3. (a) The noncontrast CT of case3 revealed multiple hypodense liver nodules. (b) The graphics of abdominal CT in the arterial dominant phase and (c) in the equilibrium phase are shown. (d) The arterial dominant phase in Gd-EOB-DTPA is shown. (e) The size of liver tumors of case3 in the arterial dominant phase of CECT have not increased when approximately 49 months has passes after the diagnosis was made. (f) In immunostaining (200×), a histological section of a hepatic specimen obtained via percutaneous liver needle biopsy in case 3 was positive for factor VIII. [file 12876_2019_1113_MOESM1_ESM.pdf]
